# Impact of the 25-Hydroxycholecalciferol Concentration and Vitamin D Deficiency Treatment on Changes in the Bone Level at the Implant Site during the Process of Osseointegration: A Prospective, Randomized, Controlled Clinical Trial

**DOI:** 10.3390/jcm10030526

**Published:** 2021-02-02

**Authors:** Jakub Kwiatek, Aleksandra Jaroń, Grzegorz Trybek

**Affiliations:** Department of Oral Surgery, Pomeranian Medical University in Szczecin, Powstańców Wielkopolskich 72/18, 70-111 Szczecin, Poland; jakubkwiatek@klinikakwiatek.pl (J.K.); aleksandra.jaron@pum.edu.pl (A.J.)

**Keywords:** oral surgery, dental implants, vitamin D, osseointegration, bone regeneration, 25-hydroxycholecalciferol

## Abstract

Introduction: The most important factor which is responsible for the positive course of implant treatment is the process of osseointegration between the implant structure and the host’s bone tissue. The aim of this study was to assess what effect the 25-hydroxycholecalciferol concentration and vitamin D deficiency treatment have on changes in the bone level at the implant site during the process of osseointegration in the mandible. Materials and Methods: The study was with 122 people qualified for implant surgery, who were assigned to three research groups (A, B, and C). Laboratory, clinical, and radiological tests were performed on the day of surgery, and after 6 and 12 weeks. The bone level in the immediate proximity of the implant was determined by radiovisiography (RVG). Results: The bone level after 12 weeks in Groups B and C was significantly higher than after 6 weeks. The bone level in the study Group B was significantly higher than in Group A. The study showed that the higher the levels of 25-hydroxycholecalciferol were observed on the day of surgery, the higher was the level of bone surrounding the implant after 6 and 12 after surgery. Conclusion: The correct level of 25-hydroxycholecalciferol on the day of surgery and vitamin D deficiency treatment significantly increase the bone level at the implant site in the process of radiologically assessed osseointegration.

## 1. Introduction

The implant–bone bond and how it is used for embedding a dental prosthesis is of key importance to implant prosthetic treatment. The process of forming of a stable, structural, and functional bond between living bone and the implant surface is called osseointegration [1,2].

In the process of osseointegration, the priority is that the implant material is not rejected by the recipient tissue, and, consequently, mechanisms leading to the production of new tissue around the implant surface are activated [3]. The success of the process is influenced by a number of factors related to both the properties of bone tissue and the material used to make the implant [4].

It is assumed that the mechanisms of intraosseous integration of the implant include osteoconduction, i.e., de novo bone formation as well as its reconstruction, the so-called remodeling [5]. In the process of bone tissue reconstruction, bone tissue alters its structure from a fibrous, plexiform repair bone with poor mechanical properties, to a trabecular structure arranged as a three-dimensional network, and then to a highly mineralized lamellar bone. In the course of continuous redevelopment, i.e., the so called “remodeling”, sites of secondary contact between the recipient tissue and the implant are formed, and new bone is formed by replacing old bone after it is resorbed by osteoclasts [6]. The mature tissue formed around the implant surface contains marrow cavities as well as blood vessels and surrounds the bone left in the bed after drilling. In the formation of new bone, during the first weeks after surgery, these fragments seem to be of significant importance, and it is suggested that they should not be removed from the bed after its preparation. Many studies have shown that various growth and differentiation factors, such as BMP and lactoferrin, enhance bone regeneration [7,8,9,10,11].

### Vitamin D

Because it participates in bone metabolic processes and controls the immune system, vitamin D is currently of particular interest to doctors who perform implant procedures [12]. It is assumed that the correct concentration of this prohormone correlates positively with the osseointegration process. Numerous studies show that vitamin D is potentially important for the process of postsurgical tissue repair, as well as integration of the implant with bone tissue, and bone homeostasis around the implant after it is loaded with a prosthetic crown [13].

Vitamin D pleiotropism is also of interest to modern dentistry, mainly as a result of its influence on bone metabolism [14,15,16,17]. Numerous bone remodeling processes that take place in the process of osseointegration depend on correct calcium and phosphate metabolism [18].

During the postsurgical phase and also after loading the implant with a prosthetic restoration, the subsequent influence of vitamin D metabolites on the processes of homeostasis with bone tissue adjacent to the implant surface, which determines the correct induction of osteoclasts and osteoblasts, is particularly important [19]. The function of vitamin D reducing inflammation around the implant is also particularly noteworthy. In addition, at the boundary between the implant and the prosthetic crown, vitamin D induces regional cells of the immune system (e.g., production of 1-alpha-hydroxylase by monocytes) [19].

According to the best of our knowledge, the literature is deficient in unambiguous results and implant treatment plans in which the level of vitamin D3 is taken into account. This was the motivation for conducting this study.

The aim of this study was to evaluate the impact of the 25-hydroxycholecalciferol concentration and vitamin D deficiency treatment on changes in the bone level at the implant site during the process of osseointegration in the mandible. The authors assume that vitamin D does increase the bone level at an implant site in the process of osseointegration assessed radiologically.

## 2. Materials and Methods

The study was conducted after obtaining the consent of the Bioethics Committee (KB-001258/18 as of 23 April 2018).

The study covered 122 people with missing teeth in the premolar or molar regions in the mandible for whom implant treatment had been suggested. The patients who qualified for the procedure were generally healthy adults, with no known no allergy to dental materials or drugs, did not take regular medications, and did not take any vitamin or mineral supplements. Recruitment of patients took place from September 2018 to February 2019.

The exclusion criteria included inappropriate bone conditions (necessity to perform augmentation procedures), general diseases, being under the age of 18 years, smoking, gingivitis, and periodontitis. All available patients meeting the inclusion and exclusion criteria, who applied to the clinic from September 2018 to February 2019, by convenience sampling, were consider to be participants for the study.

The patients were divided into 3 study groups:

1. Study Group A (*n =* 43) with <30 ng/mL of 25-hydroxycholecalciferol in blood serum, without vitamin D supplementation;
2. Study Group B (*n =* 48) with <30 ng/mL of 25-hydroxycholecalciferol in blood serum, with vitamin D supplementation;
3. Study Group C (*n =* 31) with the normal level of 25-hydroxycholecalciferol in blood serum (≥30 ng/mL).

Allocation of patients to Groups A and B was carried out using block randomization, it was assumed that 48 patients each would be assigned to Groups A and B. Five participants were excluded from Group A in the course of the study because they did not attend the check-ups on the specified date.

All the patients gave their informed consent to participate in the study and accepted the treatment plan they were shown, by confirming it with an applicable signature.

### 2.1. Qualification for Surgery

On the first visit, the medical history and physical examination were performed. The dimensions of the alveolar part of the mandible at the site where implantation was planned were assessed by means of volumetric tomography (CBCT) using the Ortophos SL 3D apparatus (2017, Dentsply Sirona, York, PA, USA). Those patients whose implantation site was characterized by an appropriate amount of horizontal and vertical bone tissue were qualified for surgery. After taking the implant diameter into account, a margin of at least 2 mm of the vestibular and lingual bone plate was kept. The position of the implant was determined using software designed for its precise site designation.

### 2.2. Description of the Surgical Procedure

All surgical procedures and control tests were performed by one operator. The implantation was performed under local, infiltrative anesthesia (2% lidocaine with 0.01 mg of epinephrine). Then, the bed was drilled according to the implant system drilling protocol using a low-speed contra-angle handpiece and an array of drill bits. The surgery area was cooled with sterile saline (0.9% NaCl).

Conical implants (MIS Implants Technologies Ltd. Bar Lev Industrial Park, BAR-LEV, Israel), with surface made by combining sandblasting and acid etching, made of grade 23 titanium (Ti 6Al4-V ELI), with conical connection and an anti-rotation, six-position index with diameter of 3.3–4.2 mm and length of 8–11.5 mm, were inserted into the bed using a contra-angle handpiece with a maximum torque of 45 Ncm. The implant was secured with a locking screw and the wound was closed with single loop sutures.

After surgery, a cold compress was applied for 30 min to the cheek skin adjacent to the region on which the operation was performed. Postsurgical recommendations included the use of an antiseptic rinse based on a 0.1% chlorhexidine solution.

### 2.3. Clinical and Laboratory Tests

The first test was performed on the day of surgery and it included assessment of the implant stability and a laboratory analysis of the 25-hydroxycholecalciferol level in blood serum. Six weeks after implantation, a radiographic image was taken, and a control examination of the level of 25-hydroxycholecalciferol was performed. Twelve weeks after surgery, before the healing screw was inserted, the success of the osseointegration process was assessed by carrying out a comparative measurement of the implant stability. A radiographic image was taken and the third examination of the 25-hydroxycholecalciferol level in blood serum was commissioned. Table 1 shows the reference levels of 25-hydroxycholecalciferol as adopted by the laboratory.

A laboratory analysis was performed three times, i.e., on the day of surgery, after six weeks, and after twelve weeks. The level of 25-hydroxycholecalciferol was monitored for a comparative analysis as well as exclusion of the possibility of achieving toxic levels in patients who took supplements. All samples were tested in the same accredited reference laboratory, according to standardized procedures, by ECLIA electrochemiluminescence on the Cobas 8000 (Roche) analyzer.

After taking into account the recommendations adopted in the study, a decision was made to supplement the study Group B with vitamin D. The group’s level of 25-hydroxycholecalciferol in blood serum was below 30 ng/mL. In light of European guidelines, the recommended dose for the treatment of vitamin D deficiencies for people over 19 years of age is 7000–10,000 IU/24 h [20,21]. The patients were instructed to take 8000 IU of vitamin D daily for the entire duration of the study.

### 2.4. Radiological Assessment of the Bone Level in the Proximity of the Implant

**Figure 1:**
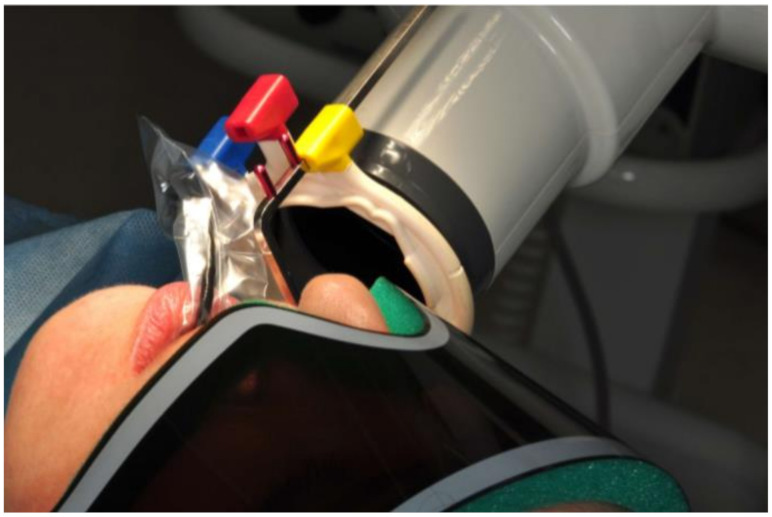
RVG images taken with the long cone paralleling technique using a positioner.

**Figure 2:**
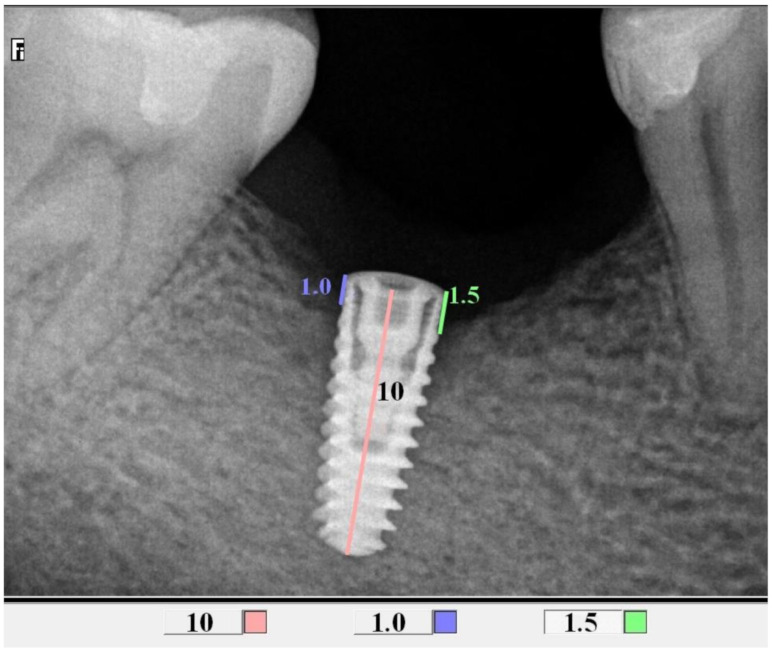
Measurements of bone loss in the proximity of the implant (DIS, dental image software).

**Figure 3:**
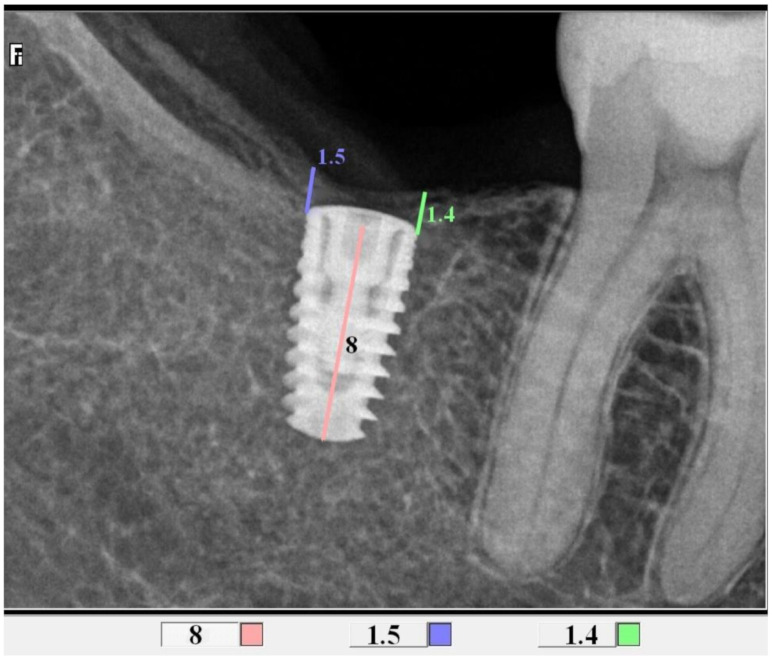
Measurements of bone growth in the proximity of the implant (DIS, dental image software).

**Figure 4:**
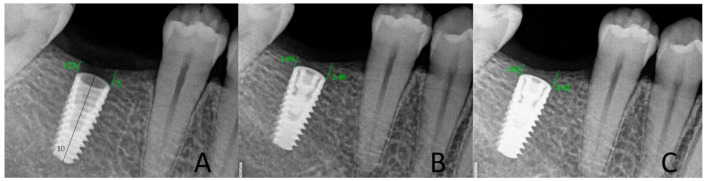
Measurements of bone growth in the proximity of the implant (DIS, dental image software). (**A**) On the day of surgery; (**B**) after 6 weeks; (**C**) after 12 weeks, in study groups.

All control tests were performed by the second operator (GT). The evaluation of bone loss/growth was performed on the basis of radiovisiography (RVG) images taken with the long cone paralleling technique using a positioner (Figure 1). The bone level in the immediate proximity of the implant was determined using the RVG image analysis. Three targeted radiographic images were compared on the day of implantation, and after six and twelve weeks. Bone tissue loss or growth were assessed using software designed for analyzing digital radiovisiography. The calculated value was the arithmetical mean of the measurements performed on both sides of the implant that were visible in the two-dimensional image (the calculations were made starting at the head of the closing screw). To enable effective calibration, a measurement was made along the long axis of the implant (a) (Figure 2, Figure 3 and Figure 4), which was related to its actual length (y) and was calculated from Formula (1) as follows:

(1) $$x=\frac{y\times a}{b}$$

where x, is bone growth, y is the implant length, a is the implant length on RVG, and b is the bone growth on RVG.

### 2.5. Statistical Analysis Methodology

Statistical analysis was performed using R software version 3.5.3 [22].

The significance level of *p* = 0.05 was assumed in the analysis. All p values lower than 0.05 were interpreted as showing the existence of significant relationships.

The Student’s *t*-test was used to compare the values of quantitative variables in two groups when normal distribution of a particular variable occurred. Otherwise, the Mann–Whitney test was used.

A comparison of the values of quantitative variables was performed using ANOVA in three or more groups in the case of normal distribution of the variable in the groups. Otherwise, the Kruskal–Wallis test was used. A post hoc analysis, after detecting statistically significant differences, was performed using the least significant differences (LSD) test if normal distribution occurred. Otherwise, the Dunn test was used.

When both quantitative variables were normally distributed, the correlations between them were analyzed using the Pearson correlation coefficient. Otherwise, the Spearman correlation coefficient was used. The strength of relationships was interpreted according to the following Hinkle’s scheme [23]:

∘ |r| ≥ 0.9, very strong relationship;
∘ 0.7 ≤ |r| < 0.9, strong relationship;
∘ 0.5 ≤|r|< 0.7, medium strong relationship;
∘ 0.3 ≤ |r| < 0.5, weak relationship;
∘ |r|< 0.3, very weak relationship (omitted).

## 3. Results

### 3.1. Characteristics of the Study Group

A total of 122 people who qualified for implant surgery were included in the study. The patients were divided into the following three groups: study Group A (*n =* 43) with 25-hydroxycholecalciferol at a level below 30 ng/mL in blood serum, consisting of 20 women and 23 men; study Group B (*n =* 48) with 25-hydroxycholecalciferol at a level below 30 ng/mL in blood serum, consisting of 22 women and 26 men receiving postsurgical vitamin D supplementation, and Group C (*n =* 31) with 25-hydroxycholecalciferol at a level of 30 ng/mL or more in blood serum, consisting of 23 women and eight men. A total of 99 implantations were performed in the molar region and 23 implantations were performed in the premolar region in the mandible. The mean age of the patients was 43.8 (±12.15). From the CBCT examination, both the length and the diameter of the implants were determined. The length values were 8 mm (*n =* 31), 10 mm (*n =* 80), and 11.5 mm (*n =* 11). The diameter values were 3.3 mm (*n =* 14), 3.75 mm (*n =* 59,) and 4.2 mm (*n =* 49). The average torque was 36.92 ± 7.77 Ncm. The average ISQ value was 78.67 ± 6.51 ISQ. Table 2 shows baseline characteristics. Table 3 shows Level of 25-Hydroxycholecalciferol on day of surgery, after 6 and 12 weeks in study groups.

### 3.2. An Analysis of Changesinf the Bone Level at the Implant Site after Six and Twelve Weeks Following Implant Surgery

In the analysis of study Groups B and C, differences in bone loss/growth at the implant site were found to be statistically significant (*p* < 0.05). It was shown that the bone level after 12 weeks was significantly higher than after 6 weeks (Table 4).

### 3.3. A Comparison of the Bone Level between the Groups at the Implant Site after Six and Twelve Weeks Following Implant Surgery

The analysis of the study groups showed statistical significance (*p* < 0.05) for differences in the bone level at the implant site after 12 weeks. In Group B it was significantly higher than in Group A (Table 5).

### 3.4. An Analysis of the 25-Hydroxycholecalciferol Concentration on the Day of Surgery and the Process of Bone Loss/Growth at the Implant Site

It was shown that in Group C the concentration of 25-hydroxycholecalciferol on the day of surgery correlated significantly (*p* < 0.05) and positively with the bone level at the implant site after 6 and 12 weeks. The higher the levels of 25-hydroxycholecalciferol were observed on the day of surgery, the higher the level of bone surrounding the implant, 6 weeks and after 12 weeks following surgery. It was shown that in Group A and B and ABC the concentration of 25-hydroxycholecalciferol on the day of surgery did not correlate significantly. (Table 6).

### 3.5. A Comparative Analysis of Changes in the Concentration of 25-Hydroxycholecalciferol between the Day of Surgery and the Sixth and Twelfth Week after Surgery and the Process of Bone Loss/Growth at the Implant Site, Six and Twelve Weeks Following Implant Surgery

The analysis showed no statistically significant correlations (*p* > 0.05) between changes of the level of 25-hydroxycholecalciferol between the day of surgery and the 6th and 12th week after surgery and bone loss/growth at the implant site 6 weeks after implant surgery.

The statistical analysis showed that, after 12 weeks following implant surgery in Group C, the bone level at the implant site correlated significantly (*p* < 0.05) and negatively with changes in the concentration of 25-hydroxycholecalciferol between the day of surgery and the sixth week. Higher bone levels after 6 weeks were observed in Group C in patients whose level of 25-hydroxycholecalciferol was changed to a lesser degree. This data is presented in Table 7.

### 3.6. A Comparative Analysis of Changes in the Concentration of 25-Hydroxycholecalciferol between the Day of Surgery and the Sixth and Twelfth Week after Surgery as Well as Changes of the Bone Level at the Implant Site, Six and Twelve Weeks Following Implant Surgery

It was shown that bone loss/growth at the implant site correlated significantly (*p* < 0.05) and positively in study Group B with changes in the 25-hydroxycholecalciferol concentration between the day of surgery and the sixth week. The greater the change in the 25-hydroxycholecalciferol concentration between the day of surgery and the sixth week, the greater the change in the bone level at the implant site.

The change of the bone level at the implant site in Group C correlated significantly (*p* < 0.05) and negatively with the change in the 25-hydroxycholecalciferol concentration between the day of surgery and the 6th and 12th week. The greater the change was in the 25-hydroxycholecalciferol concentration in Group C, the lower was bone growth at the implant site. This data is presented in Table 8.

## 4. Discussion

The influence of vitamin D on a number of sequential factors in the process of implant–bone integration seems to be of significant importance in each of its stages [24]. Therefore, it is extremely important to study the phenomenon of a connection formed between bone tissue and the surface of a titanium implant and find correlations between the 25-hydroxycholecalciferol concentration in blood serum and the course of osseointegration.

Because the processes of bone remodeling are very dynamic in the period of actual osseointegration, it is necessary to obtain the correct concentration of vitamin D3 metabolites in blood serum. After implantation, primary stabilization is obtained. The remodeling of bone tissue that occurs over the next few months leads to integration of the implant with bone, which is called secondary stabilization. During osseointegration, the influence of calcitriol on calcium and phosphate metabolism, as well as on the processes of activation and differentiation of osteoblasts and osteoclasts, is of significant importance. It was found, among others, that osteoclasts (osteoclast cells), which, by the way, have no vitamin D receptors, are formed as a result of the fusion of about 5 to 10 precursor cells influenced by vitamin D. Osteoblasts (osteogenic cells) have that receptor, and the bone modeling process occurs through osteoclasts contacting osteoblasts via RANK and RANKL receptors [25].

In the context of the continuing success of implant treatment, maintaining the optimal condition of tissues at the implant site is as important as obtaining stabilization. The cause of implant disintegration after achieving osseointegration may be gradual loss of bone tissue around the implant, which results from the inflammation in the region [26]. Hence, it seems particularly important to monitor the bone level in the proximity of the implant after treatment is completed. If the implant is overloaded or no appropriate hygiene standards are met, peri-implantitis may occur [27]. As a result, the implant may be lost. In the postsurgical period, the degree of bone tissue growth or loss at the implant site may be an indicator of treatment success. The criterion established for long-term implantation success is the loss of a maximum 0.2 mm of bone at the implant site per year [28].

An analysis of the effects of 25-hydroxycholecalciferol on the bone tissue in the mandible was conducted by Kawakami et al. [29]. The study was performed using an animal model. In rats, tooth movement was stimulated by orthodontic treatment. Some of the rats were topically administered a vitamin D supplement. Then, tests were performed to check whether the supplementation induced formation of the alveolar process or not. A test performed after 14 days showed a significantly higher growth of bone tissue in rats that received vitamin D supplements. Unfortunately, the level of vitamin D in their blood serum was not determined during the study. The study was conducted for fourteen days only. Our own research study showed that the bone level at the implant site started to change significantly after 12 weeks.

The relationships demonstrated in the study are consistent with the results of the analysis by Hong et al. [30], carried out on a group of dogs, which were administered vitamin D supplements after implantation in the mandible, and 4 weeks after surgery the bone level at the implant site was measured. As a result, stimulating effects of supplementation on bone tissue were found. Dogs that were given vitamin D showed an increase in the bone level at the implant site [30]. Unfortunately, the authors did not determine the levels of vitamin D in canine blood serum prior to the study. The dogs were randomly divided into two groups. Apart from vitamin D, calcium was also supplemented, which may have influenced the results of the analysis.

Dvorak et al. [31] conducted a study on rats whose vitamin D concentration in blood serum was modified with dietary factors. In one group, products with vitamin D were totally excluded. In a second group, standard food was provided, and in a third group vitamin D was excluded in the first stage of the study, and then 2400 IU/kg of body weight were administered. The study confirmed that in the group of rats with vitamin D excluded from the diet, the level of 25-hydroxycholecalciferol in blood serum decreased in relation to the level before the study. Moreover, the highest bone tissue resorption and the smallest bone contact surface with the implant were found in that group. Unfortunately, the studies were carried out on an animal model, and the study groups consisted of only 15 rats.

An international team led by Naito [32] obtained different results. The researchers assessed the effect of 1,25-dihydroxyvitamin D administered directly to the implant surface on the bone level and the surface of bone to implant contact. Unfortunately, the study was performed on a group that was more than three times smaller than in our own study on an animal model, and another form of vitamin D was used. During the study, the level of vitamin D in blood serum was not determined, and the analysis was carried out on rabbits.

Our own study on patients showed a significant correlation between changes of the bone level at the implant site and the level of 25-hydroxycholecalciferol in blood serum. The analysis showed a statistical significance for the difference in bone levels around the implant, 12 weeks following surgery. In the group which received deficiency treatment (Group B), it was significantly higher than in the group which took no vitamin D supplements (Group A).

It should be taken into consideration that our research also has some limitations. We had to believe the patients that they were taking 8000 IU of Vitamin D daily (24 h) because they were taking the drug themselves at home. From the study Group A, five people were removed from the study because they did not show up for the control tests.

Although the studies were carried out after the period of greatest exposure to sunlight, it cannot be ruled out that patients were exposed to sunlight to varying degrees.

In view of the scientific reports and the analysis carried out in our own study, we suggest that 25-hydroxycholecalciferol is a potential factor that stimulates the growth of bone tissue at implant sites. It is worth paying attention to the benefits resulting from determining the concentration of vitamin D metabolite on the process of implant osseointegration and soft tissue repair. On the basis of the growth of bone tissue observed and assessed radiographically in the group which took vitamin D supplements, we conclude that monitoring the 25-hydroxycholecalciferol concentration along with radiological assessment of the bone level is a simple and minimally invasive method of assessing the process of osseointegration at the intraosseous implant site. Such an algorithm could be introduced into the record of the assessment of the results of implant treatment.

## 5. Conclusions

The correct level of 25-hydroxycholecalciferol on the day of surgery and vitamin D deficiency treatment have a significant influence on the increase in the bone level at the implant site during the process of osseointegration assessed radiologically.

## Figures and Tables

**Table 1:** 25-Hydroxycholecalciferol reference values adopted by the laboratory.

Concentration	Reference Range [ng/mL]
Deficiency	<20
Low	20–30
Optimal	30–50
High	50–100
Potentially toxic	100–150
Toxic	>150

**Table 2:** Baseline characteristics.

Feature		Study Group A (*n* = 43)	Study Group B (*n* = 48)	Study Group C (*n* = 31)	Groups ABC (*n* = 122)	*p* *
Age (years)	mean ± SD	45.79 ± 12.78	42.52 ± 12.16	43 ± 11.24	43.8 ± 12.15	0.406
	Median	46	40.5	40	43	P
	Quartiles	37.5–57.5	33–53.5	36–50	34–53.75	
Implant diameter	3.3 mm	4 (9.30%)	2 (4.17%)	8 (25.81%)	14 (11.48%)	<0.001
	3.75 mm	14 (32.56%)	26 (54.17%)	19 (61.29%)	59 (48.36%)	F
	4.2 mm	25 (58.14%)	20 (41.67%)	4 (12.90%)	49 (40.16%)	
Implant lenth	8 mm	8 (18.60%)	13 (27.08%)	10 (32.26%)	31 (25.41%)	0.71
	10 mm	30 (69.77%)	31 (64.58%)	19 (61.29%)	80 (65.57%)	F
	11.5 mm	5 (11.63%)	4 (8.33%)	2 (6.45%)	11 (9.02%)	
Sex	Woman	20 (46.51%)	22 (45.83%)	23 (74.19%)	65 (53.28%)	0.026
	Man	23 (53.49%)	26 (54.17%)	8 (25.81%)	57 (46.72%)	chi2
Region of implantation	premolars	8 (18.60%)	7 (14.58%)	8 (25.81%)	23 (18.85%)	0.46
	Molars	35 (81.40%)	41 (85.42%)	23 (74.19%)	99 (81.15%)	chi2
Bone type (Misch)	D1	3 (6.98%)	4 (8.33%)	5 (16.13%)	12 (9.84%)	0.454
	D2	28 (65.12%)	36 (75.00%)	20 (64.52%)	84 (68.85%)	F
	D3	12 (27.91%)	7 (14.58%)	6 (19.35%)	25 (20.49%)	
	D4	0 (0.00%)	1 (2.08%)	0 (0.00%)	1 (0.82%)	
Osseointegration	+	40 (93.02%)	47 (97.92%)	31 (100.00%)	118 (96.72%)	0.366
	−	3 (6.98%)	1 (2.08%)	0 (0.00%)	4 (3.28%)	F

chi2, chi-squared test; F, Fisher’s exact test; P, normal distribution parametric analysis, ANOVA + post-hoc analysis (Fisher’s LSD); Kruskal-Wallis test + post hoc analysis (Dunn test); SD, standard deviation; * *p* significance level; *n*, number of patients.

**Table 3:** Level of 25-Hydroxycholecalciferol on day of surgery, after 6 and 12 weeks in study groups.

25-Hydroxycholecalciferol (ng/mL)		Day of Surgery	After 6 Weeks	After 12 Weeks	*p* *
Study group A	Mean ± SD	22.32 ± 4.42	22.06 ± 6.4	22.27 ± 7.33	0.926
	Median	23.4	22.2	22	NP
	Quartiles	21.5–25.05	18.95–25.05	16.5–28.65	
Study group B	Mean ± SD	17.95 ± 6.07	35.14 ± 9.69	38.87 ± 10.64	<0.001
	Median	17.45	35.85	38.65	NP
	Quartiles	13.95–23.8	27.12–40.38	33.52–44.32	C > B > A
Study group C	Mean ± SD	40 ± 9.19	40.24 ± 10.08	38.73 ± 9.52	0.02
	Median	36.7	38	36.4	NP
	Quartiles	33.45–45.9	32.3–45.4	32.75–45	B,A > C
Groups A B C	Mean ± SD	25.09 ± 11.04	31.82 ± 11.5	32.98 ± 12.17	0.002
	Median	23.9	30.4	33.15	NP
	Quartiles	17.72–28.67	24.1–39.27	23.9–39.98	C,B > A

NP, deficiency in normality of distribution; Frierman test + post-hoc analysis (Wilcoxon signed-rank test with Bonferroni correction); * *p* significance; level.

**Table 4:** A comparison of the bone level at the implant site after 6 and 12 weeks in the study groups (A, B, and C).

Bone Level (mm)		After 6 weeks	After 12 weeks	*p* *
Study group A	Mean ± SD	0.06 ± 0.48	0.08 ± 0.92	0.232 NP
	Median	0	0.2	
	Quartiles	0–0.3	–0.05–0.65	
Study group B	Mean ± SD	0.25 ± 0.51	0.53 ± 0.77	<0.001 NP
	Median	0.25	0.65	
	Quartiles	0–0.6	0.1–1.02	
Study group C	Mean ± SD	0.29 ± 0.53	0.48 ± 0.74	0.008 P
	Median	0	0.3	
	Quartiles	0–0.55	0–0.9	

P, normal distribution of differences, Student’s *t*-test for dependent (repeated) measurements; NP, deficiency in normality of distribution of differences, Wilcoxon test for dependent (repeated) measurements; * *p* significance; level.

**Table 5:** A comparison of the bone level between the study groups at the implant site after 6 and 12 weeks (A, B, and C).

Bone Level (mm)		Study Group A	Study Group B	Study Group C	*p* *
After 6 weeks	Mean ± SD	0.06 ± 0.48	0.25 ± 0.51	0.29 ± 0.53	0.146 NP
	Median	0	0.25	0	
	Quartiles	0–0.3	0–0.6	0–0.55	
After 12 weeks	Mean ± SD	0.08 ± 0.92	0.53 ± 0.77	0.48 ± 0.74	0.028
	Median	0.2	0.65	0.3	NP
	Quartiles	−0.7	0.1–1.02	0–0.9	B > A

NP, deficiency in normality of distribution, non-parametric analysis, Kruskal–Wallis test + post hoc analysis (Dunn test); * *p* significance; level.

**Table 6:** A comparison of the 25-hydroxycholecalciferol concentration on the day of surgery and bone loss/growth after 6 and 12 weeks.

Bone Level		Correlation with 25-Hydroxycholecalciferol on the Day of Surgery			
		Correlation Coefficient	*p* *	Direction of Relationship	Strength of Relationship
Study group C	After 6 weeks	0.364	0.044 NP	Positive	Weak
	After 12 weeks	0.429	0.016 NP	Positive	weak

NP, deficiency in normality of distribution of at least one of the variables correlated, Spearman’s correlation coefficient; * *p* significance level.

**Table 7:** A comparison of changes in the 25-hydroxycholecalciferol concentration on the day of surgery, 6 and 12 weeks following surgery, and bone loss/growth around the implant after 6 and 12 weeks.

25-Hydroxycholecalciferol		Correlation with the Bone Level after 6 Weeks				Correlation with the Bone Level after 12 Weeks			
		Correlation Coefficient	p *	Direction of Relationship	Strength of Relationship	Correlation Coefficient	p *	Direction of Relationship	Strength of Relationship
Study Group C	Change between the day of surgery and the 6th week	−0.315	0.084 NP	---	---	−0.439	0.013 NP	negative	weak
	change between the day of surgery and the 12th week	−0.185	0.318 NP	---	---	−0.337	0.063 NP	---	---
	Change between the 6th week and the 12th week	0.146	0.432 NP	---	---	0.097	0.603 NP	---	---

NP, deficiency in normality of distribution of at least one of the variables correlated, Spearman’s correlation coefficient; * *p*, significance level.

**Table 8:** A comparison of changes in the 25-hydroxycholecalciferol concentration on the day of surgery, 6 and 12 weeks following surgery, and changes of the bone level at the implant site between the 6th and 12th week.

25-Hydroxycholecalciferol		Correlation with Changes of the Bone Level between the 6th and 12th Week			
		Correlation Coefficient	*p* *	Direction of Relationship	Strength of Relationship
Study Group B	Change between the day of surgery and the 6th week	0.302	0.037 NP	positive	weak
	Change between the day of surgery and the 12th week	0.094	0.527 NP	---	---
	Change between the 6th week and the 12th week	−0.173	0.24 NP	---	---
Study Group C	Change between the day of surgery and the 6th week	−0.421	0.018 NP	negative	weak
	Change between the day of surgery and the 12th week	−0.384	0.033 NP	negative	weak
	Change between the 6th week and the 12th week	−0.015	0.938 P	---	---

P—normal distribution of distribution variables correlated, Pearson’s correlation coefficient, NP—deficiency in normality of distribution of at least one of the variables correlated, Spearman’s correlation coefficient, * *p*—significance level.

## Data Availability

Data available on request due to restrictions eg privacy or ethical.
